# Highly efficient export of a disulfide‐bonded protein to the periplasm and medium by the Tat pathway using CyDisCo in *Escherichia coli*

**DOI:** 10.1002/mbo3.1350

**Published:** 2023-03-08

**Authors:** Klaudia Arauzo‐Aguilera, Mirva J. Saaranen, Colin Robinson, Lloyd W. Ruddock

**Affiliations:** ^1^ School of Biosciences University of Kent Canterbury UK; ^2^ Faculty of Biochemistry and Molecular Medicine University of Oulu Oulu Finland

**Keywords:** CyDisCo, disulfide bond, *Escherichia coli*, periplasm, signal peptide, Tat pathway

## Abstract

High‐value heterologous proteins produced in *Escherichia coli* that contain disulfide bonds are almost invariably targeted to the periplasm via the Sec pathway as it, among other advantages, enables disulfide bond formation and simplifies downstream processing. However, the Sec system cannot transport complex or rapidly folding proteins, as it only transports proteins in an unfolded state. The Tat system also transports proteins to the periplasm, and it has significant potential as an alternative means of recombinant protein production because it transports fully folded proteins. Most of the studies related to Tat secretion have used the well‐studied TorA signal peptide that is Tat‐specific, but this signal peptide also tends to induce degradation of the protein of interest, resulting in lower yields. This makes it difficult to use Tat in the industry. In this study, we show that a model disulfide bond‐containing protein, YebF, can be exported to the periplasm and media at a very high level by the Tat pathway in a manner almost completely dependent on cytoplasmic disulfide formation, by other two putative Tat SPs: those of MdoD and AmiC. In contrast, the TorA SP exports YebF at a low level.

## Graphical abstract

Most studies related to Tat secretion have used the well‐studied Tat‐specific TorA signal peptide, but this signal peptide also tends to induce degradation of the protein of interest, resulting in poor yields. In this study, we show that YebF can be exported to the periplasm and media at a very high level by the Tat pathway in a manner almost completely dependent on the CyDisCo system for disulfide bond formation in the cytoplasm by two other Tat signal peptides: those of MdoD and AmiC.

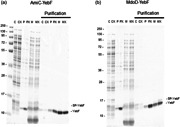

## 1 INTRODUCTION

High‐value heterologous proteins produced in *Escherichia coli* that contain disulfide bonds (DSB) are almost invariably targeted to the periplasm via the general secretory (Sec) pathway by means of a cleavable N‐terminal signal peptide (SP) (Mirzadeh et al., 2020). This guides newly synthesized proteins through the SecYEG membrane channel in an unfolded state. Once across the membrane, the SP is cleaved and proteins fold in the periplasm, acquiring DSBs where appropriate (Kleiner‐Grote et al., 2018). This protein export approach offers several advantages to produce therapeutic proteins, such as (i) it enables disulfide bond formation, which only occurs in the periplasm in wild‐type cells, (ii) it facilitates protein isolation from the relatively small periplasmic proteome, (iii) allows control of the nature of the N‐terminus of the mature protein, and (iv) minimizes exposure to cytoplasmic proteases (Karyolaimos & de Gier, 2021).

Some proteins, however, fold too rapidly for the Sec system to handle, or require co‐factor insertion in the cytoplasm, thereby precluding translocation via the Sec system. The twin‐arginine translocation (Tat) pathway offers a potential alternative method of localizing proteins to the periplasm and, unlike Sec, this system transports fully folded proteins. As with Sec substrates, Tat substrates are synthesized with N‐terminal SPs, but these contain Tat‐specific determinants including the presence of a highly conserved twin‐arginine motif (reviewed by Natale et al., 2008).

An additional, and indeed unique, feature of the Tat pathway is its in‐built proofreading mechanism that can detect structurally incorrect substrates and reject them for export. This proofreading capability could allow for a more homogeneous product to be produced in the periplasm (reviewed by Frain et al., 2019). Furthermore, the recent development of TatExpress (TE) strains, that over‐express the *tatABC* genes (encoding the Tat system) from the chromosome boosts the industrial potential of this pathway (Browning et al., 2017). However, this quality control poses problems for the export of disulfide‐bonded proteins, because such proteins often only obtain a native conformation after the formation of DSB. CyDisCo strains (cytoplasmic disulfide bond formation in *E. coli*), express a catalyst of disulfide bond formation, usually the sulfhydryl oxidase Erv1p, and a catalyst of disulfide isomerization, usually human protein disulfide isomerase (PDI). This system facilitates DSB formation in the cytoplasm of wild‐type *E. coli* and may therefore allow the efficient production of DSB‐containing proteins in the cytoplasm before their export via the Tat pathway (Matos et al., 2014).

The efficiency of protein secretion varies depending on the host strain, signal sequence, and the type of protein to be secreted (Freudl, 2018). There are at least 29 SPs in the *E. coli* genome that contain a twin‐arginine motif characteristic of proteins exported via the Tat pathway. However, many of these SPs are not completely Tat‐specific and can lead to the secretion of the protein of interest (POI) via Sec, Tat, or both depending on the POI, strain, or media used. They are sometimes termed promiscuous SPs (Bendtsen et al., 2005; Tullman‐Ercek et al., 2007). In contrast, the TorA SP, an *E. coli* Tat SP derived from pre‐trimethylamine *N*‐oxide (TMAO) reductase (TorA), is a very well‐studied SP that is reported to be Tat‐specific (Blaudeck et al., 2001; Jack et al., 2004; Lee et al., 2006). However, the use of the TorA SP also tends to induce degradation of the protein of interest, for unknown reasons. This results in low yields of some POI, as seen in the absence of precursor forms in many export studies (e.g., Alanen et al., 2015).

While it has been shown that Tat can export some DSB‐ed proteins at high levels in fed‐batch fermentation studies (e.g., hGH; Guerrero Montero et al., 2019a) and shake‐flask studies (Alanen et al., 2015; Browning et al., 2017; DeLisa et al., 2003; Matos et al., 2014; Tullman‐Ercek et al., 2007), it has yet to be determined whether it can efficiently export a protein that requires DSB formation for correct folding. In this study, we used YebF, a 10.8 kDa *E. coli* protein of unknown function that contains a single DSB. It has been previously reported that recombinant YebF is secreted by laboratory strains of *E. coli* into the extracellular medium after first being translocated into the periplasm by the Sec‐system (Zhang et al., 2006). A wide variety of proteins, including N‐glycosylated protein domains, are readily secreted into the growth medium via fusion with YebF (Fisher et al., 2011; Haitjema et al., 2014).

Here, we report that YebF can be exported to the periplasm and media by the Tat pathway in an almost completely CyDisCo‐dependent manner. The use of TorA SP results in low yields, consistent with other POI previously examined. However, we show that two other Tat SPs, namely MdoD and AmiC, direct very high levels of protein export. While both may be capable of directing export by Sec, expression in TatExpress strains results in an increase in export flux and expression without CyDisCo or in ΔTat cells inhibits export, which all indicate that export is largely carried out by Tat.

## 2 MATERIALS AND METHODS

All chemicals used in this study were supplied by Fisher Scientific (Thermo Fisher Scientific Inc.), Sigma (Sigma‐Aldrich), or Formedium unless otherwise stated.

### 2.1 Cloning

The desired gene fragments were obtained as synthetic genes from GeneArt or by PCR from *E. coli* genomic DNA and cloned with restriction digestion and ligation into a modified pET23‐based vector with a pTac promoter replacing the T7 promoter (Gaciarz et al., 2016). The vector design allows for the incorporation of a C‐terminal hexahistidine tag (‐Leu‐Glu‐6xHis) into the expressed protein. For YebF without the signal sequence, a vector incorporating both N‐ (Met‐6xHis‐) and C‐terminal hexahistidine tags was used. For constructs with signal sequences, the signal sequence plus the first four amino acids of the mature protein was added to YebF, to increase the likelihood of efficient processing of the signal sequence by the signal peptidase. The net effect of this is that each purified mature construct differs slightly from the other, resulting in mass differences by mass spectrometry and small mobility differences in SDS‐PAGE. The gene inserts in the plasmids made were fully sequenced before use (see Table 1 for plasmid names and details).

**Table 1:** Strains and constructs used in this study.

Strain/plasmid	Description	Source/reference
BL21	*E. coli* B F^–^ *dcm ompT lon hsdS*(r_B_^–^ m_B_^–^) *gal*	Agilent
BL21 TatExpress	BL21 carrying a pTac promotor upstream of tatABCD	Browning et al. (2017)
ΔTatABCDE (ΔTat)	MC4100 strain (*Ara* ^*R*^, *F2 araD139 DlacU169 rpsL150 relA1 flB5301 deoC1 ptsF25 rbs* ^*R*^) lacking tat*ABCDE* genes, *Ara* ^*R*^	Wexler et al. (2000)
pMJS289	YebF (A22‐R118)^a^	This study
pMJS285	AmiC (M1‐Q35)‐YebF (A22‐R118)^b^	This study
pMJS284	MdoD (M1‐D36)‐YebF (A22‐R118)^b^	This study
pMJS288	TorA (M1‐A43)‐YebF (A22‐R118)^b^	This study
pMJS205	CyDisCo: Erv1p and PDI	Gaciarz et al. (2016)
pAG82	empty	Gaciarz et al. (2016)

^a^ With N‐terminal Met‐6xHis and C‐terminal ‐Leu‐Glu‐6xHis ‐tags.

^b^ With C‐terminal ‐Leu‐Glu‐6xHis ‐tag.

### 2.2 Expression

Plasmids with the gene of interest together with the plasmid containing the CyDisCo components (pMJS205) or empty plasmid (pAG82) were cotransformed into chemically competent *E. coli* cells and spread onto lysogeny broth (LB) agar plates supplemented with 35 µg/mL of chloramphenicol and 100 µg/mL of ampicillin for selection. After overnight incubation at 37°C these were used to inoculate 2–5 mL of LB media supplemented with 2 g/L of glucose, 35 µg/mL of chloramphenicol, and 100 µg/mL of ampicillin. These starter cultures were grown 6 h, or overnight for ΔTat experiments, at 30°C, 250 rpm (2.5 cm radius of gyration), and were used to seed the cultures in a 1:100 ratio.

Expression tests to screen for optimal SP were carried out for the constructs in 24 deep well plates (DWP). The constructs were expressed alone or co‐expressed with CyDisCo components in *E. coli* BL21 in 3 mL per well of terrific broth autoinduction media (AIM—Terrific Broth Base including Trace elements, Formedium) supplemented with 0.8% glycerol, 35 µg/mL of chloramphenicol and 100 µg/mL of ampicillin. The DWP was covered with air permeable membrane (Thomson) and the cultures were grown at 30°C, 250 rpm, and harvested after 24 h. The cells were collected by centrifugation at 3220*g* for 20 min at 4°C and resuspended in 3 mL of 50 mM sodium phosphate pH 7.4, 20 μg/ml DNase, 0.1 mg/mL egg white lysozyme. After 10 min incubation, the resuspended cultures were frozen at −20°C. Cells were lysed by freeze‐thawing.

Main cultures with selected constructs in either BL21 or BL21 TatExpress cells were grown in 100 mL flasks with 10 mL culture in each flask. The flasks were covered with oxygen‐permeable membranes (Thomson) to ensure proper oxygenation of the cultures, grown at 30°C, 250 rpm for 24 h, and harvested for fractionation.

For ΔTat experiments, cultures were grown in LB media at 30°C, 250 rpm in shake flasks until the OD_600_ of the cultures reached approximately 0.5 and were then induced with 50 µM isopropyl ß‐D‐1‐thiogalactopyranoside for 2 h before harvesting.

### 2.3 Fractionation of the cells

For the fractionation of the cells, the PureFrac fractionation protocol was used (Malherbe et al., 2019). For purification, ethylenediamine tetraacetic acid (EDTA) was not added to any of the buffers. Apart from the periplasm and cytoplasm, medium samples were also recovered (same volume in all cultures). In ΔTat experiments, the 1× phosphate‐buffered saline wash of the cells and the separation of the cytoplasm and insoluble fraction was not carried out to avoid extra manipulation of this cell line due to its fragility. Samples were prepared for sodium dodecyl sulphate polyacrylamide gel electrophoresis (SDS‐PAGE) analysis in reducing conditions.

### 2.4 Purification of cytoplasmic, periplasmic, and medium samples, SDS‐PAGE analysis, and western blot (WB) analysis

Purification of hexahistidine‐tagged proteins was performed by standard immobilized metal affinity chromatography using HisPur Cobalt resin (Thermo Scientific) under native conditions. For 3 mL cultures from 24 DWP, IMAC was performed using 0.2 mL resin in small gravity feed columns. The resin was washed with 2 × 2 mL of water and equilibrated with 2 × 2 mL of 50 mM phosphate buffer (pH 7.4). Cell lysates on 24 DWP were cleared by centrifugation (3220*g*, 20 min, 4°C) and loaded onto the columns. The columns were rinsed with 2 mL of 50 mM phosphate buffer (pH 7.4), washed with 4 × 2 mL of wash buffer (50 mM sodium phosphate, 10 mM imidazole, 300 mM sodium chloride; pH 7.4), and then rinsed with 2 mL of 50 mM sodium phosphate (pH 7.4) before elution with 3 × 0.2 mL of 50 mM sodium phosphate, 50 mM EDTA (pH 7.4). For 10 mL cultures, the same protocol was used with the following changes: medium samples were 1:2 diluted (total volume 10 mL), periplasmic and cytoplasmic fractions were diluted in 2.5 mL of 200 mM sodium phosphate buffer and made up to 10 ml with water to reduce the salt concentration. Samples were prepared for SDS‐PAGE analysis and 10 μL were loaded in 4–20% Criterion™ TGX™ Precast Midi Protein Gel, 26 well (BioRad).

For the detection of proteins by WB analysis the method as detailed in Guerrero‐Montero, Dolata, et al. (2019) was performed, with the exception that was transferred to the polyvinylidene fluoride‐membrane (GE Healthcare) by rapid semi‐dry transfer using the Invitrogen Power Blotter XL System according to the manufacturer's instructions.

### 2.5 Mass spectrometry

The theoretical oxidized monoisotopic molecular weight (*M*_theorOx_) of the His‐tag YebF constructs in dalton (Da) was calculated using the ExPaSy Compute pI/Mw ‐tool (Gasteiger et al., 2005) (Table 2). The molecular weights of purified protein samples were measured by electrospray ionization mass spectrometry combined with liquid chromatography (LC‐ESI‐MS) using a Q Exactive Plus Mass Spectrometer. The protein samples were mixed with trifluoroacetic acid (TFA) to a final concentration of 0.5% before analysis. For N‐ethylmaleimide (NEM)‐trapped samples the protein was incubated with 20 mM NEM in 50 mM phosphate buffer pH 7.3 with 6 M guanidine‐HCl for 10 min and quenched with 0.5% TFA before analysis.

**Table 2:** Theoretical oxidized and experimental molecular weights of the proteins in this study.

Construct	Location	Number of cysteine	*M*_theorOx_ (Da)	*M*_exp_ (Da)	Δ mass
YebF no SP	Cytoplasm	2	12,937	12,985	48
AmiC‐YebF	Periplasm		12,506	12,538	32
	Medium		12,506	12,538	32
MdoD‐YebF	Periplasm		12,509	12,541	32
	Medium		12,509	12,541	32
TorA‐YebF	Periplasm		12,320	12,352	32
	Medium		12,320	12,352	32

*Note*: The theoretical monoisotopic molecular weight for oxidized (*M*_theorOx_) His‐tag YebF constructs in dalton (Da) were calculated using the ExPaSy Compute pI/Mw tool (Gasteiger et al., 2005). The experimental molecular weight (*M*_exp_) was determined by mass spectrometry. The same masses were obtained with NEM treatment. The results suggest both cysteines are in a disulfide bond in the YebF constructs analyzed.

## 3 RESULTS

### 3.1 Folding of YebF is CyDisCo dependent without its native SP and with the AmiC, MdoD, and TorA SPs

In this study, the first aim was to demonstrate the potential use of other Tat SPs in place of the well‐known but non‐ideal TorA SP (Alanen et al., 2015). We also sought to test whether a CyDisCo‐dependent protein could be exported at high rates by the Tat system; *E. coli* TatExpress cells have been shown to export high levels of human growth hormone (hGH) but while this protein contains two DSB, they are not essential for proper folding and the Tat system can efficiently export the protein in its reduced state (Alanen et al., 2015 and references therein). Tat can only transport fully folded proteins from the cytoplasm to the periplasm, so we hypothesized that a disulfide‐containing protein that required disulfide formation to reach a native state, for example, one that was dependent on CyDisCo in our system, would be a good model protein to test the true capabilities of the Tat system. Based on its reported use as a transport mechanism for secretion to the medium (Fisher et al., 2011; Haitjema et al., 2014; Zhang et al., 2006), we initially chose a small disulfide bonded *E. coli* protein as a test protein—YebF.

**Figure 1:**
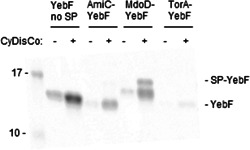
Purification of soluble YebF protein without its native SP and with AmiC, MdoD, and TorA SPs in the absence/presence of CyDisCo. Coomassie‐stained Criterion^TM^ TGX gels showing the purified soluble YebF (≈12 kDa) in BL21 wild‐type in 2 mL rich‐autoinduction media at 30°C from 24DWP. All the constructs show a CyDisCo dependency for the production of YebF soluble protein (marked as “‐YebF”). MdoD‐YebF construct shows uncleaved YebF (shown as “SP‐YebF”) as well as mature protein (marked as “YebF”) in the presence of CyDisCo. SP, signal peptide.

In the absence of an SP (Figure 1, “YebF no SP”), the folding of YebF showed a strong CyDisCo dependence, with purified YebF being far more abundant in the +CyDisCo cells, indicating that CyDisCo improves folding to a significant extent. However, some soluble protein was present in the absence of CyDisCo indicating that the protein can fold to a low extent without CyDisCo. YebF was then tested with the widely used TorA Tat‐dependent SP (construct denoted TorA‐YebF) along with AmiC and MdoD SPs as potential alternative Tat SPs (referred to as AmiC‐YebF and MdoD‐YebF, respectively). In all cases, YebF was expressed with a 6xHis tag on the C‐terminus. To achieve efficient SP cleavage, all SPs were fused with four more N‐terminal residues to YebF, resulting in differences between the mature YebF dependent on the SP used.

For both the AmiC‐YebF and MdoD‐YebF constructs there was a strong dependency on CyDisCo to produce a soluble protein (Figure 1), indicating that the protein was probably secreted via the Tat pathway rather than via the Sec pathway. If the proteins were exported by Sec, they would be unfolded until they reached the periplasm where they would rapidly acquire their DSBs, and CyDisCo would not influence their folding. An extra band in the gel (labeled SP‐YebF) was observed for MdoD‐YebF, which probably represents uncleaved SP‐YebF (the POI with the SP still attached). This suggests that for this construct in these conditions, export to the periplasm was limiting. In contrast to the results with AmiC‐YebF and MdoD‐YebF, the TorA‐YebF construct produced very low levels of soluble protein in the presence of CyDisCo and hardly detectable levels in the absence of CyDisCo. While the possibility of issues connected with messenger RNA stability linked to the sequence encoding the SP, initiation of translation initiation, and so forth cannot be excluded, it is likely that this difference reflects inefficient transport by Tat and the protein being degraded in the cytoplasm as observed with other constructs bearing the TorA SP (Alanen et al., 2015).

### 3.2 TatExpress cells export much more YebF to the periplasm than standard BL21 cells with AmiC and MdoD SPs

We next assessed the export of the four constructs by fractionating cell samples into the cytoplasm (C), periplasm (P), and medium (M) to discover where and how in the cell or medium this protein was targeted in the presence of CyDisCo. The experiments were carried out in both a standard BL21 strain and the BL21 TatExpress strain which has been engineered for higher levels of Tat‐dependent export (Browning et al., 2017). We reasoned that if the AmiC‐YebF and MdoD‐YebF constructs were exported primarily by Tat, we might observe higher levels of export in the TatExpress cells, if the export was limiting, as observed with hGH (Browning et al., 2017; Guerrero Montero, Richards, et al., 2019), and that total yields might increase if the AmiC or MdoD SP would act similar to the TorA SP and target non‐exported protein for degradation.

**Figure 2:**
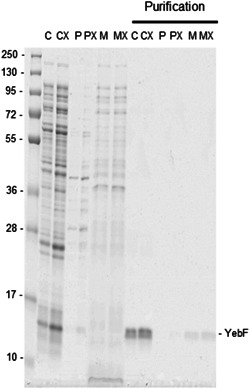
Expression of YebF control without SP in BL21 and BL21 TatExpress cells with CyDisCo. Coomassie‐stained Criterion^TM^ TGX gel of cytoplasmic, periplasmic fractions and medium samples and purifications (C, P, M) from BL21 and BL21 TatExpress cells (CX, PX, MX) expressing YebF without SP (marked as “YebF”). Samples were collected after 24 h of growth in terrific broth‐based autoinduction media at 30°C in shake flasks and processed immediately. Samples from the same subcellular fraction are comparable between strains, but not between different subcellular compartments. Cytoplasm was diluted in 750 μL buffer. Periplasm was diluted in 400 μL buffer. For all medium samples, a 5 mL culture was recovered. Purifications of all cell fractions and medium are comparable among fractions and among strains. Some POI is visible in medium purification probably due to cell lysis. The representative gel from triplicate experiments is shown.

The control experiment showed that YebF when expressed without an SP, remains in the cytoplasm when expressed in both BL21 and BL21 TatExpress (Figure 2; lanes “C” denote cytoplasmic fraction; “X” denotes TatExpress cells in this and subsequent Figures). No YebF was visible in the periplasm (Figure 2 lanes P, PX). IMAC purification of the protein from fractions confirmed the cytoplasmic localization (lanes denoted “Purification”), with a small amount of YebF being purified from the medium, most likely due to cell lysis.

**Figure 3:**
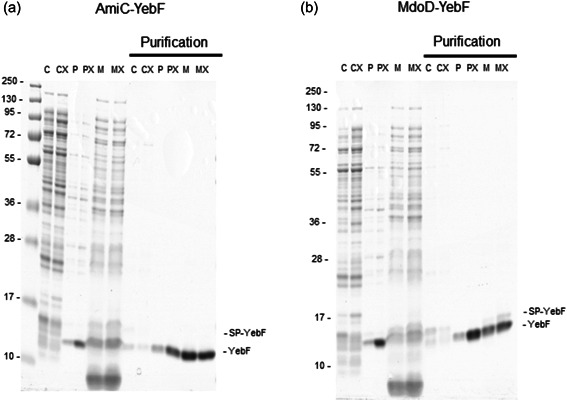
Export of AmiC‐ and MdoD‐ YebF in BL21 and BL21 TatExpress cells with CyDisCo. Coomassie‐stained Criterion^TM^ TGX gel of cytoplasmic, periplasmic fractions and medium samples and purifications (C, P, M) from BL21 and BL21 TatExpress cells (CX, PX, MX) expressing AmiC‐YebF, MdoD‐YebF, and TorA‐YebF (marked as “‐YebF” and “SP‐YebF”). Samples were analyzed after 24 h of growth in terrific broth‐based autoinduction media at 30°C in shake flasks. (a) AmiC‐YebF fractionation and purification of BL21 and BL21 TatExpress cells for comparison. (b) MdoD‐YebF fractionation and purification of BL21 and BL21 TatExpress for comparison. Samples from the same subcellular fraction are comparable between strains, but not between different subcellular compartments. Cytoplasm was diluted in 750 μL buffer. Periplasm was diluted in 400 μL buffer. Purifications of all cell fractions and medium are comparable among fractions and among strains. For all medium samples, a 5 mL culture was recovered. The representative gel from triplicate experiments is shown.

**Figure 4:**
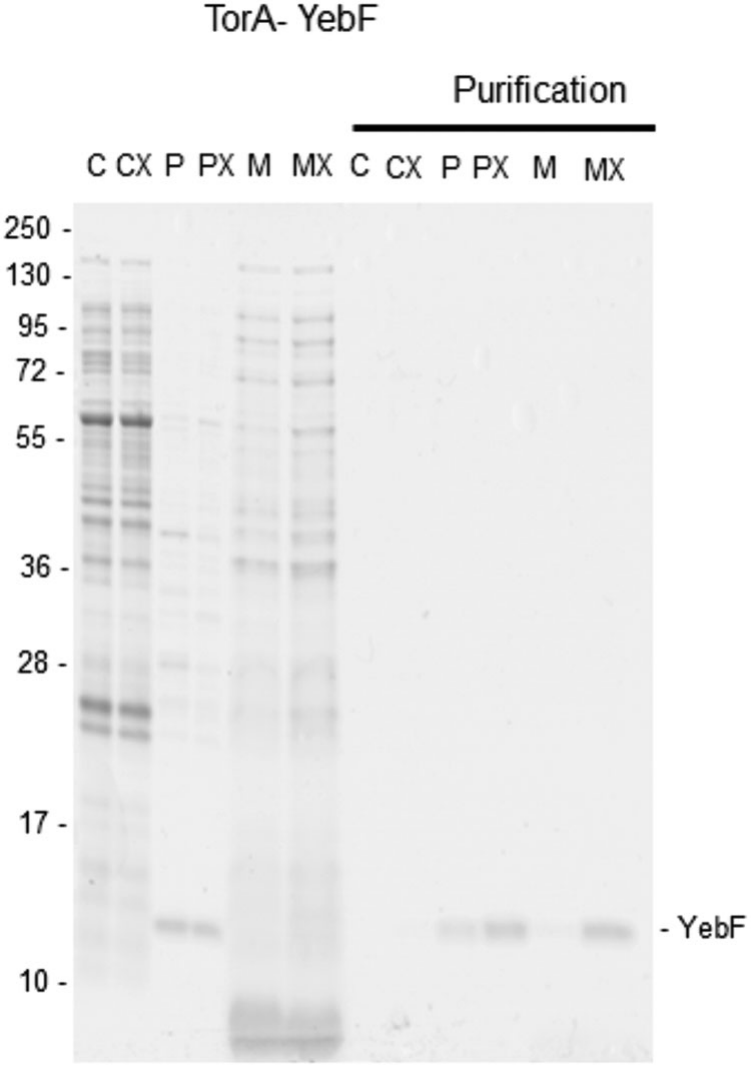
Export of TorA‐YebF in BL21 and BL21 TatExpress cells with CyDisCo. Coomassie‐stained Criterion™ TGX gel of cytoplasmic, periplasmic fractions and medium samples and purifications (C, P, M) from BL21 and BL21 TatExpress cells (CX, PX, MX) TorA‐YebF (marked as “‐YebF”). Samples were analyzed after 24 h of growth in terrific broth‐based autoinduction media at 30°C in shake flasks. Samples from the same subcellular fraction are comparable between strains, but not between different subcellular compartments. Cytoplasm was diluted in 750 μL buffer. Periplasm was diluted in 400 μL buffer. Purifications of all cell fractions and medium are comparable among fractions and among strains. For all medium samples, a 5 mL culture was recovered. The representative gel from triplicate experiments is shown.

Since YebF without a signal sequence could be efficiently folded by CyDisCo but was retained in the cytoplasm, we then examined if YebF could be exported to the periplasm and, if so, whether this export was increased in TatExpress cells when using the AmiC‐, MdoD‐ and TorA SPs (Figures 3 and 4). The constructs were expressed in BL21 and BL21 TatExpress and the cells fractionated into cytoplasm, periplasm, and medium (C, P, M) with YebF purified by IMAC in the “Purification” panels.

In both constructs and strains, the periplasmic fraction is relatively clean of cytoplasm cross‐contaminants as judged by the low levels or absence of major cytoplasmic proteins. Importantly, YebF was the most abundant periplasmic protein after expression of AmiC‐YebF and MdoD‐YebF, even in standard BL21 cells, and its abundance increased significantly in TatExpress cells (Figure 3a,b lanes PX). IMAC purification of YebF confirmed that the export of both proteins is particularly efficient in TatExpress cells. Purification from the cytoplasmic fractions showed a very faint duplex band for AmiC‐YebF and a slightly more prominent duplex for the MdoD‐YebF construct, which probably represent the SP‐ and mature forms of YebF. In purifications from periplasm fractions, only the mature form was seen for both constructs. In purifications from the media again the duplex was observed for the MdoD‐YebF construct, consistent with some of the protein in the media deriving from cell lysis as per the control YebF without a SP (Figure 2). For both proteins, but in particular, for MdoD‐YebF the total amount of YebF observed increased in TatExpress cells (Figure 3b), suggesting that like the TorA SP, nonsecreted YebF retaining the SP might be targeted for degradation in the cytoplasm (a process which will probably be SP, strain, media, and protein‐dependent).

To compare the efficiency of the export of these two newly proposed Tat‐specific SPs to the well‐characterized TorA SP, TorA‐YebF was also expressed in BL21 and BL21 TatExpress, and the cells fractionated into cytoplasm, periplasm, and medium (C, P, M) with YebF again purified by IMAC in the “Purification” panels. YebF was also exported to the periplasm and to the medium (Figure 4). While YebF protein was again the most abundant protein in the periplasm, indicating efficient Tat‐dependent export, it was considerably less than for the MdoD‐ or AmiC‐SP constructs (Figure 1 and compare Figure 3 vs. Figure 4). Since the TorA SP is reported to be highly Tat‐specific (Tullman‐Ercek et al., 2007), this export must be by the Tat pathway, which in turn means that the DSB‐ed protein is being exported.

Together, the results suggest a very efficient export by the Tat pathway to the periplasm, which was higher for AmiC‐YebF and MdoD‐YebF compared with TorA‐YebF. In addition, purified yields of YebF from the medium fractions were high. This may come partially from lysis but probably arises mainly from the translocation of YebF from the periplasm to the medium by an unknown mechanism (Zhang et al., 2006).

### 3.3 Examination of Tat‐dependence

The CyDisCo dependency for AmiC‐, MdoD‐, and TorA‐YebF folding and export (Figure 1) and the enhancement of export to the periplasm in TatExpress cells (Figure 3), both are consistent with the hypothesis that the predominant pathway used for all three constructs must be Tat, as Sec will only transport proteins in an unfolded state.

To examine this further, we expressed our constructs in a strain (ΔTat) that lacks the *tatABCDE* genes that encode the Tat apparatus. In this strain, any periplasmic export must be via Sec. ΔTat is a relatively fragile strain that tends to lyse more easily than wild‐type strains in many growth conditions, has a higher level of proteases, is difficult to subfractionate, and is more sensitive to stress (Harrison et al., 2005; Sargent et al., 1998).

**Figure 5:**
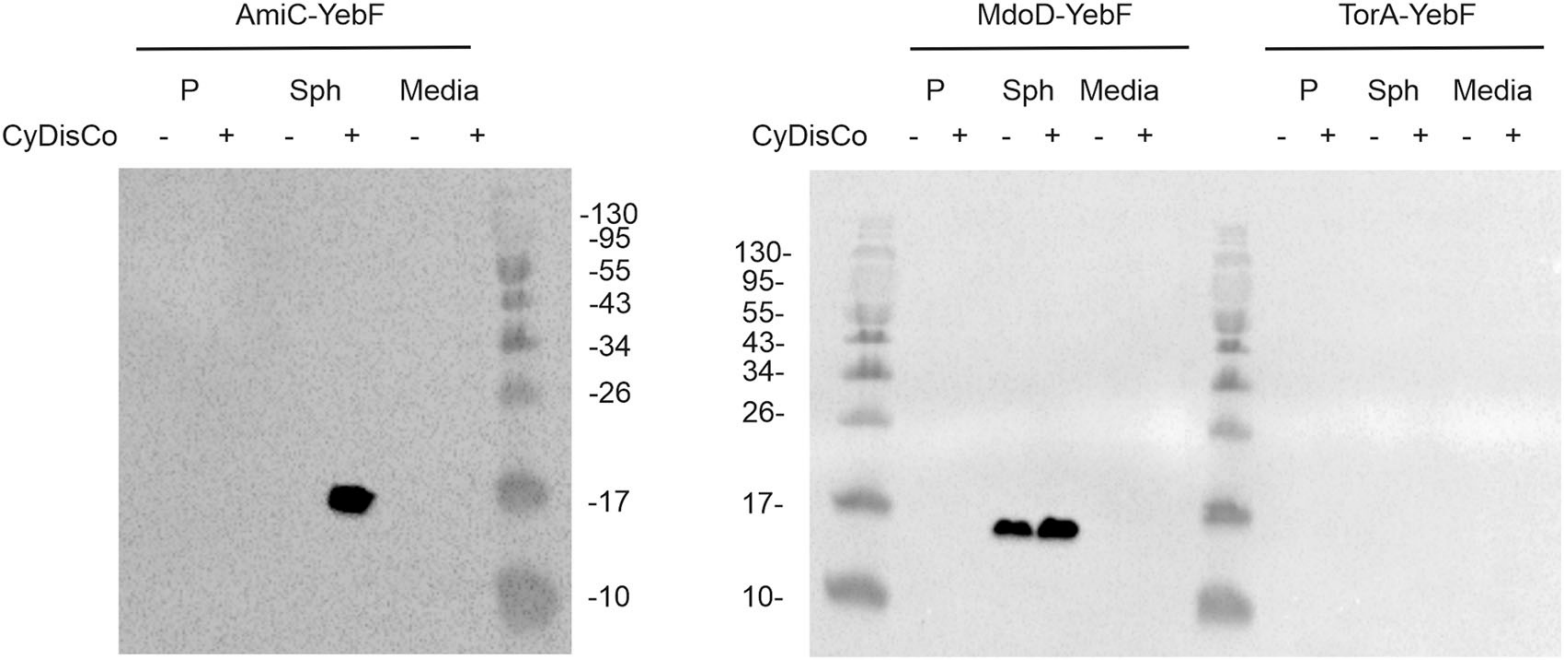
Expression of YebF SPs constructs in ΔTat strain with and without CyDisCo. western blot analysis of 15% SDS‐PAGE gel of periplasmic (P), spheroplast (Sph) fractions, and medium (M) samples expressing YebF SP constructs. Samples were analyzed 2 h postinduction (50 μM IPTG) in LB media at 30°C in shake flasks. Samples from the same subcellular fraction are comparable between strains, but not between different subcellular compartments. Spheroplast was diluted in 750 μL buffer. Periplasm was diluted in 400 μL buffer. For all medium samples, 500 μL culture was recovered. Note the different SDS‐PAGE gel type results in different mobilities c.f. Figures 1, 2, 3, 4. LB, lysogeny broth; SDS‐PAGE, sodium dodecyl sulphate polyacrylamide gel electrophoresis; SP, signal peptide.

To minimize cross‐contamination between fractions, cells were grown in a rich medium that only resulted in low‐density cultivation and induced for short times before harvesting. This resulted in lower levels of protein expression and hence WB analysis, using an antibody against the C‐terminal his‐tag, was used to examine the subcellular localization. Analysis of medium samples suggested very low levels of lysis in these growth conditions (Figure 5).

While no protein was observed for TorA‐YebF under these conditions, whether CyDisCo was expressed or not, consistent with it being a poor signal sequence, strong bands were observed for both AmiC‐YebF and MdoD‐YebF when CyDisCo was present. These bands were observed only in the spheroplast fractions and not in the periplasm or media fractions, consistent with our hypothesis that the export of YebF is predominantly via Tat.

While AmiC‐YebF appears to be completely CyDisCo dependent under these expression conditions (similar to the strong CyDisCo‐dependence observed in Figure 1), MdoD‐YebF shows less CyDisCo‐dependence than observed in the previous expression conditions (compare Figure 5 and Figure 1). This effect may arise from the solubilization of folding intermediates promoted by fusion partners (including potentially SPs), with different fusions resulting in different solubilization effects and/or degradation rates. The differences observed between experiments (Figure 5 and Figure 1) may arise from differences in expression conditions (media and time of induction) or cells (including higher levels of proteases in ΔTat cells) or the very different levels of protein produced.

### 3.4 YebF control and SP‐YebF constructs are folded and correctly cleaved from SPs in the periplasm and media

A final experiment to verify the folding and the correct cleavage of the SPs of the constructs in the periplasm and medium was carried out by subjecting the purified proteins to ESI‐Mass Spectrometry. The theoretical monoisotopic molecular weight for oxidized (*M*_theorOx_) YebF with no SP purified from the cytoplasm is 12,937 Da and we observed an experimental molecular weight (*M*_exp_) of 12,985 Da, which showed a difference of 48 Da (see Table 2). The *M*_theorOx_ of AmiC‐YebF purified from the periplasm and medium is 12,506 Da and we observed an *M*_exp_ of 12,538 Da, which showed a difference of 32 Da. For MdoD‐YebF purified from the periplasm and medium, the *M*_theorOx_ is 12,509 Da and we observed an *M*_exp_ of 12,541 Da, which also showed a difference of 32 Da. For TorA‐YebF purified from the periplasm and medium, the *M*_theorOx_ is 12,320 Da and we observed an *M*_exp_ of 12,352 Da, which also showed a difference of 32 Da. Overall, analysis by ESI‐MS confirmed that YebF purified from the cytoplasm (in the YebF no SP construct only), periplasm, and medium had the expected molecular weight, consistent with the cleavage of the respective SP when the POI is exported from the cytoplasm to the periplasm and its two cysteines form of a disulfide bond. The presence of free cysteines in the constructs was further evaluated by treating the samples with NEM before mass spectrometric analysis. NEM‐trapped samples would be expected to show an addition of 125 Da in the molecular weight of the protein for each free cysteine modified. None of the samples analyzed showed any increase in the mass after NEM treatment, implying that none contained free thiols (Nguyen et al., 2011; Saaranen et al., 2010). The change of mass of 48 Da (for YebF no SP) and 32 Da for the other three constructs are due to the oxidation of methionine in the purified proteins (three and two oxygen molecules, respectively). The handling, time of storage, and/or repeatedly freeze‐thawed, were responsible for this phenomenon (Grassi & Cabrele, 2019) (Table 2).

## 4 DISCUSSION

*E. coli* production platforms are extensively used for the production of biotherapeutics, but current platforms have limitations in terms of the types of protein that they can handle. Typically, target proteins are refolded from inclusion bodies or targeted to the periplasm where they fold to a native state, the latter being the less time‐consuming and labor‐intensive approach (Bhatwa et al., 2021). Tat‐based platforms offer significant advantages for the production of some molecules, but the system has not been validated using a wide range of proteins, especially proteins that require disulfide bonding to fold correctly, and there is a clear need to find an alternative to TorA as the commonly used Tat specific SP. The vast majority of export studies by the Tat pathway have been carried out using the TorA SP although it is known that it can cause POI degradation in the cytoplasm (Blaudeck et al., 2003) and inclusion body formation (Jong et al., 2017). The TorA SP has been shown to export a small number of proteins with high efficiency, but none of these examples required disulfide bonding to occur in the cytoplasm before translocation by Tat (Alanen et al., 2015).

Here, we set out to compare the export of YebF to the periplasm of *E. coli via* the Tat pathway with different SPs: the AmiC and MdoD SPs have been reported to be able to go through both the Sec and Tat pathway (Tullman‐Ercek et al., 2007) whereas the TorA SP is reported to be Tat specific.

We examined the export to the periplasm (and medium) of YebF with different SPs in the presence and absence of CyDisCo which is required for YebF to efficiently reach a native state in the cytoplasm. In wild‐type cells, YebF was exported to the periplasm and medium by the classical Tat SP TorA, but yields were relatively low—as is often reported for this SP. The use of TatExpress cells increased export to the medium, while no YebF could be observed in a ΔTat strain, as expected with an SP that cannot target proteins through the Sec pathway.

Generally, the results for AmiC SP and MdoD SP mirrored those of the TorA SP, with two exceptions. First, the periplasmic and medium yields of YebF in wild‐type and TatExpress cells were far higher than for TorA, that is, they appeared to be much more efficient SPs. Second, using WB analysis in ΔTat cells protein could be detected and was only observed in the spheroplast fraction without showing any export to the periplasm or media. While this implies that both SPs are Tat‐specific under these expression conditions, we do not believe that either is completely Tat‐specific. Rather we believe that while Tat is the normal/predominant secretion pathway for the AmiC and MdoD SPs, Sec‐dependent secretion can occur. The trigger for promiscuity for SPs is not known, nor is the potential link between promiscuity and the protein being exported (or how efficiently it folds).

The choice of the POI for this study was not arbitrary. Apart from the desired CyDisCo dependency of the protein of choice, YebF is an intriguing POI. YebF with its native SP is used as a “passenger” protein linker to export transgenic proteins to the medium by an unknown mechanism. This discovery gives the possibility of linking more disulfide‐bonded difficult‐to‐express proteins to these two efficient and Tat‐dependent SPs for easy recovery in the extracellular medium, assuring the correct folding with CyDisCo of the target protein and a maximized yield when using TatExpress cells.

In conclusion, the AmiC and MdoD SPs appeared to allow efficient secretion of a disulfide bond containing protein from the cytoplasm of *E. coli via* Tat. While these signal sequences may not be completely Tat‐specific, the data suggests the majority of the YebF is exported via Tat. As they are far more efficient than the TorA SP, they are probably more suitable for large‐scale protein production than the reportedly more rigorously specific Tat‐SP.

## AUTHOR CONTRIBUTIONS

**Klaudia Arauzo‐Aguilera**: Formal analysis (lead); investigation (lead); visualization (lead); writing—original draft (lead). **Mirva J. Saaranen**: Formal analysis (supporting); investigation (supporting); methodology (equal); supervision (equal); writing—review and editing (equal). **Colin Robinson**: Funding acquisition (equal); supervision (equal); writing—review and editing (equal). **Lloyd W. Ruddock**: Conceptualization (equal); funding acquisition (equal); methodology (equal); supervision (equal); writing—review and editing (equal).

## CONFLICT OF INTEREST STATEMENT

A patent for the production system used to make the protein using sulfhydryl oxidases in the cytoplasm of *E. coli* is held by the University of Oulu: Method for producing natively folded proteins in a prokaryotic host (Patent number 9238817; date of patent January 19, 2016). Inventor: Lloyd W. Ruddock.

## ETHICS STATEMENT

None required.

## Data Availability

All data supporting the conclusions of this study are included in the article.
